# Long noncoding RNA LBX2-AS1-modulated miR-4766-5p regulates gastric cancer development through targeting CXCL5

**DOI:** 10.1186/s12935-020-01579-z

**Published:** 2020-10-12

**Authors:** LiPan Peng, ZeZhong Chen, GuangChuan Wang, ShuBo Tian, Shuai Kong, Tao Xu, XiaoHua An, YueZhi Chen

**Affiliations:** 1 Department of Gastrointestinal Surgery, Shandong Provincial Hospital Affiliated to Shandong First Medical University, 250021 Jinan, China; 2 Department of General Surgery, No. 1 People’s Hospital of NingYang County, Taian, 271400 China; 3 Department of Gastroenterology, Shandong Provincial Hospital Affiliated to Shandong First Medical University, Jinan, 250021 China; 4 Department of Surgery, Shandong Provincial Hospital Affiliated to Shandong First Medical University, Jinan, 250021 China

**Keywords:** GC, LBX2-AS1, miR-4766-5p, CXCL5

## Abstract

**Background:**

Long noncoding RNAs (LncRNAs) have been reported to critically regulate gastric cancer (GC). Recently, it was reported that LBX2 antisense RNA 1 (LBX2-AS1) is abnormally expressed in GC. However, the role of LBX2-AS1 in the malignancy of GC is worth further discussion.

**Methods:**

Quantitative real-time polymerase chain reaction (qRT-PCR) was used to determine the LBX2-AS1, miR-4766-5p and C-X-C motif chemokine (CXCL5) expression in GC tissues and cells. Dual-luciferase reporter assay was applied to examine the target relationship between LBX2-AS1 and miR-4766-5p or miR-4766-5p and CXCL5. Cell counting kit-8 (CCK-8) and Transwell assays were used to detect cell proliferation, migration and invasion rates. The protein expression of CXCL5 was confirmed using western blot. The RNA pull down experiment was used to verify the specificity of LBX2-AS1 and miR-4766-5p on BGC-823 and SGC-7901 cells.

**Results:**

LBX2-AS1 was up-regulated in GC tissues and cells, and its knockdown suppressed proliferation, migration and invasion of GC cells. While, overexpression of LBX2-AS1 increased proliferation and increased CXCL5 mRNA level. CXCL5 improved cell proliferation, migration and invasion of GC cells. LBX2-AS1 could bind to miR-4766-5p to regulate CXCL5 expression. Overexpression of CXCL5 overturned those effects of miR-4766-5p in GC cells. RNA Pull down shown that BGC-823 and SGC-7901 cells, miR-4766-5p specifically binds to LBX2-AS1.

**Conclusions:**

In short, this study demonstrated that LBX2-AS1 promoted proliferation, migration and invasion through up-regulation CXCL5 mediated by miR-4766-5p in GC. The LBX2-AS1/miR-4766-5p/CXCL5 regulatory axis provides a theoretical basis for the research on lncRNA-directed therapeutics in GC.

## Background

Gastric cancer (GC) is a one of the most common malignant tumors, and its morbidity ranks the first cancer in China [1]. Although the early diagnosis of GC as well as the level of surgical techniques and chemoradiotherapy has been significantly improved, the incidence of GC is still rising steadily and becoming younger [2, 3]. Therefore, it is urgent to explore the molecular mechanism of GC development to provide theoretical basis for the prevention of GC.

Long non-coding RNA (lncRNA) is a class of functional RNA molecules with transcript length of > 200 nucleotides [4]. Recently, studies have found that lncRNAs are widely involved in almost all physiological and pathological processes of the body, and is closely related to the occurrence and development of many tumors [5]. LBX2 antisense RNA 1 (LBX2-AS1), transcribed from the intron of chromosome 2p13.1, was a newly discovered lncRNA which was initially reported to act as a tumor promoter [6]. Studies have shown that LBX2-AS1indicates poor prognosis and promotes cell proliferation and metastasis through Notch signaling in non-small cell lung cancer [7]. In addition, abnormal expression of LBX2-AS1 is associated with poor prognosis of GC [8].

MicroRNAs (miRNAs) are a class containing 19 to 25 endogenous non-coding small molecule single-stranded RNA, which is widely found in eukaryotes and highly conserved. miRNAs are complementary to the bases of the 3′ non-coding regions of the target mRNA to promote the degradation of the target mRNA or inhibit its translation, thereby regulating the expression of the target gene and affecting the cell proliferation, differentiation, and apoptosis processes. LncRNAs can serve as the sponge of miRNA, down-regulate the expression of miRNA, and finally change the expression of miRNA target protein at the post-transcriptional level. As a relatively new miRNA, miR-4766-5p has been reported that plasma miR-4766-5p levels are significantly down-regulated in breast cancer [9]. It was also reported that miR-4766-5p suppressed serine/threonine kinase PAK2 to inhibit tumorigenic ability of colorectal cancer [10]. Meanwhile, Wei et al. suggested that miR-4766-5p targeted NKAP to inhibit the proliferation and metastasis of GC cells [11]. Therefore, exploring its mechanism of action can provide more theoretical basis for the treatment of GC.

As a member of the ELR + CXC chemokine family, C-X-C motif chemokine 5 (CXCL5) is an inflammatory mediator cells which recently has been determined to play a core role in some cancer. For example, elevated levels of CXCL5 were detected in human NSCLC that was related to the vascularity of these tumors [12]. Ando et al. research showed that CXCL5 could promote pancreatic cell migration and invasion by mediating the necroptosis [13]. Recently, Liu et al. reported that overexpression of CXCL5 dramatically attenuated the suppressive effects of cell proliferation, migration and invasion induced by ROR-α-1 overexpression in hepatocellular carcinoma [14]. In addition, CXCL5 also has been determined to be associated with late stage of GC [15]. Therefore, the expression of CXCL5 is closely related to the occurrence of GC.

Our study aimed to investigate the mechanism of LBX2-AS1 in GC progress. Function assays indicated that the decreased proliferation and metastasis of GC cells in response to silenced LBX2-AS1. Bioinformatics analysis and dual luciferase reporter assay was certified the interaction among LBX2-AS1, miR-4766-5p and CXCL5. Rescue assays were performed to prove the functional effects of LBX2-AS1/miR-4766-5p/CXCL5 axis in GC. All the findings are conductive to comprehend the biological functions and molecular mechanisms of LBX2-AS1 in GC progression.

## Materials and methods

### Clinical samples

78 GC patients were recruited from the Shandong Provincial Hospital Affiliated to Shandong First Medical University. Tumor samples and adjacent tissue were collected and immediately stored at -80 °C until used. The Ethics Committee of Shandong Provincial Hospital Affiliated to Shandong First Medical University approved this study, and written informed consents were acquired from all enrolled patients.

### Cell culture and transfection

Three GC cell lines (MKN-45, BGC-823 and SGC-7901) and the human gastric mucosal epithelial cell line (GES-1) were all obtained from the Shanghai Cell Bank of the Chinese Academy of Sciences (Shanghai, China) and seeded into 1640 culture medium containing 10% fetal bovine serum (FBS, Gibco), 100 IU/ml penicillin, and 100 IU/ml streptomycin in 5% CO_2_ incubator at 37 °C and 95% humidity. Small interfering RNA (siRNA) against LBX2-AS1 (si-LBX2-AS1-1 or 2) and its control (si-NC), pcDNA and pcDNA-LBX2-AS1 overexpression vector (LBX2-AS1) or pcDNA-CXCL5 overexpression vector (CXCL5), miR-4766-5p mimics (miR-4766-5p), miR-4766-5p inhibitor and matched negative controls (miR-inhibitor-NC) were obtained from Guangzhou RiboBio Co., Ltd (Guangzhou, China) and then Lipofectamine 2000 (Invitrogen, Thermo Fisher Scientific) was used to transfer them following the manufactures’ instructions.

### Overexpression of LBX2-AS1 cells

Overexpression of the truncated intracellular form of LBX2-AS1 in GC cell lines (BGC-823 and SGC-7901) was achieved using the plasmid pEGFP-N1 (Generay, Shanghai, China). To eliminate endotoxin contamination, all plasmids were prepared using an Endo-free Plasmid Mini Kit II (Omega, San Carlos, CA, United States). Transient transfection was performed with FuGENE 6 Transfection Reagent (Promega, Sunnyvale, CA, United States). Overexpression of LBX2-AS1 was confirmed with dual-endonuclease digestion and sequencing.

### Dual-luciferase reporter assay

In brief, partial fragments of LBX2-AS1 and CXCL5 3’UTR containing miR-4766-5p binding sites were subcloned into the psiCHECK-2 luciferase vector (Promega) to produce The wild-type (WT) LBX2-AS1-3ʹ-UTR (LBX2-AS1-WT) and the wild-type (WT) CXCL5-3ʹ-UTR (CXCL5-WT) reporters, respectively. Also, the mutant LBX2-AS1-3ʹ-UTR (LBX2-AS1-MUT) and the mutant CXCL5-3ʹ-UTR (CXCL5-MUT) reporters with mutant miR-4766-5p binding sites were constructed. Luciferase assay was performed the firefly luciferase 48 h post-transfection.

### Cell counting kit-8 (CCK-8) assay

The cultured cells (about 10^4^/96-well) were cultured for 24 h after transfection. Then, 10 µL CCK-8 solution (C0041, Beyotime Institute of Biotechnology, Shanghai, China) was added to each well and cultured for 4 h. The absorbance was measured with a spectrophotometer (Bio-Rad) at 450 nm to measure cells proliferation.

### Transwell assay

Transwell assay (Corning, NY, USA) was used to detect the cell migration and invasion abilities following the manufacturer’s protocol. In short, after transfected, about 1 × 10^5^ cells were seeded in an 8 µm polycarbonate membrane filter with or without Matrigel (BD Biosciences, CA, USA) in addition to 100 µl serum-free medium (Thermo Fisher Scientific) to detect the ability of cell migration and invasion in the upper chamber, and the lower chamber added 600 µl medium containing 10% FBS. After 24 h, the membrane was removed and stained at room temperature with 0.1% crystal violet for 15 min. An inverted microscope was further used to count the number of migrated and invasive cells.

### qRT-PCR

Total RNA of tissues and cells were extracted using Trizol reagent (Invitrogen, Carlsbad, CA) and qRT-PCR was performed with the SYBR Green PCR kit (Takara, Otsu, Japan) following the manufacturer’s protocol. TaqMan microRNA assays (Thermo Fisher Scientific) were used to measure miR-4766-5p level with U6 small nuclear RNA (U6-snRNA) as the internal control. The relative expressions of LBX2-AS1 and CXCL5 were calculated with GAPDH as the internal control. The primers for LBX2-AS1, CXCL5 and GAPDH were listed as below: LBX2-AS1, Forward: 5′- AGT TTG TCC CAG GTT TGG CA -3′, Reverse: 5′- CAT GCC AGG GTC CTT GTT CT -3′; CXCL5, Forward: 5′-TGT GCA ATT AAC AAA GCT ACT GCA A-3′, Reverse: 5′- AGG CAT CTA AAA AGC TCA GCA ATG-3′; GAPDH, Forward: 5′- CTG GGC TAC ACT GAG CAC C-3′, Reverse: 5′- AAG TGG TCG TTG AGG GCA ATG-3′.

### RNA pull down assay

Bio-miR-4766-5p-wt, bio-miR-4766-5p-mut and negative control were constructed in BGC-823 and SGC-7901 cells, and pulldown experiments were undertaken, followed by qRT-PCR to detect LBX2-AS1 enrichment. A total of 1 × 10^7^ GC cells were harvested, lysed and sonicated. The miR-4766-5p probe was used for incubation with C-1 magnetic beads (Life Technologies) at 25 °C for 2 h to generate probe-coated beads. Cell lysate with miR-4766-5p probe or oligo probe was incubated at 4 °C for one night. After washing with wash buffer, the RNA mix bound to the beads was eluted and extracted with an RNeasy Mini Kit (QIAGEN) for qRT-PCR.

### Western blot

The process of western blotting analysis was conducted as described by precious studies [16]. The protein of sample was extracted by using RIPA Lysis Buffer containing PMSF (Solarbio, China). The protein concentration in each extract was determined using a BCA Protein Quantification Kit (Beyotime, Shanghai, China). Samples of total protein were separated by 10% SDS-PAGE, and the protein bands were transferred onto PVDF membranes, which were subsequently blocked with 5% skim milk for 2 h. Subsequently, the membranes were incubated with primary antibodies against CXCL5 (1:1000, ab9802, Abcam, MA, USA) or GAPDH (1:2000, ab181602, Abcam, MA, USA) overnight at 4 °C and then incubated with secondary antibody labeled with HRP (Abcam) for 1 h at 37 °C after washed with TBST for four times. ECL chromogenic substrate was used to visualize the bands, and the Image J software was used to determine the gray value of each band.

### Statistical analysis

All statistical analyses were analyzed using the SPSS 21.0 statistical software (SPSS, Chicago, IL) in conjunction with Prism 8.0 software (GraphPad, San Diego, CA). All experimental results were expressed as mean ± SD. The data were compared by one-way ANOVA, and a P-value < 0.05 was considered statistically to be statistically significant.

## Results

### LBX2-AS1 was highly expressed in GC tissue and cells

**Fig. 1:**
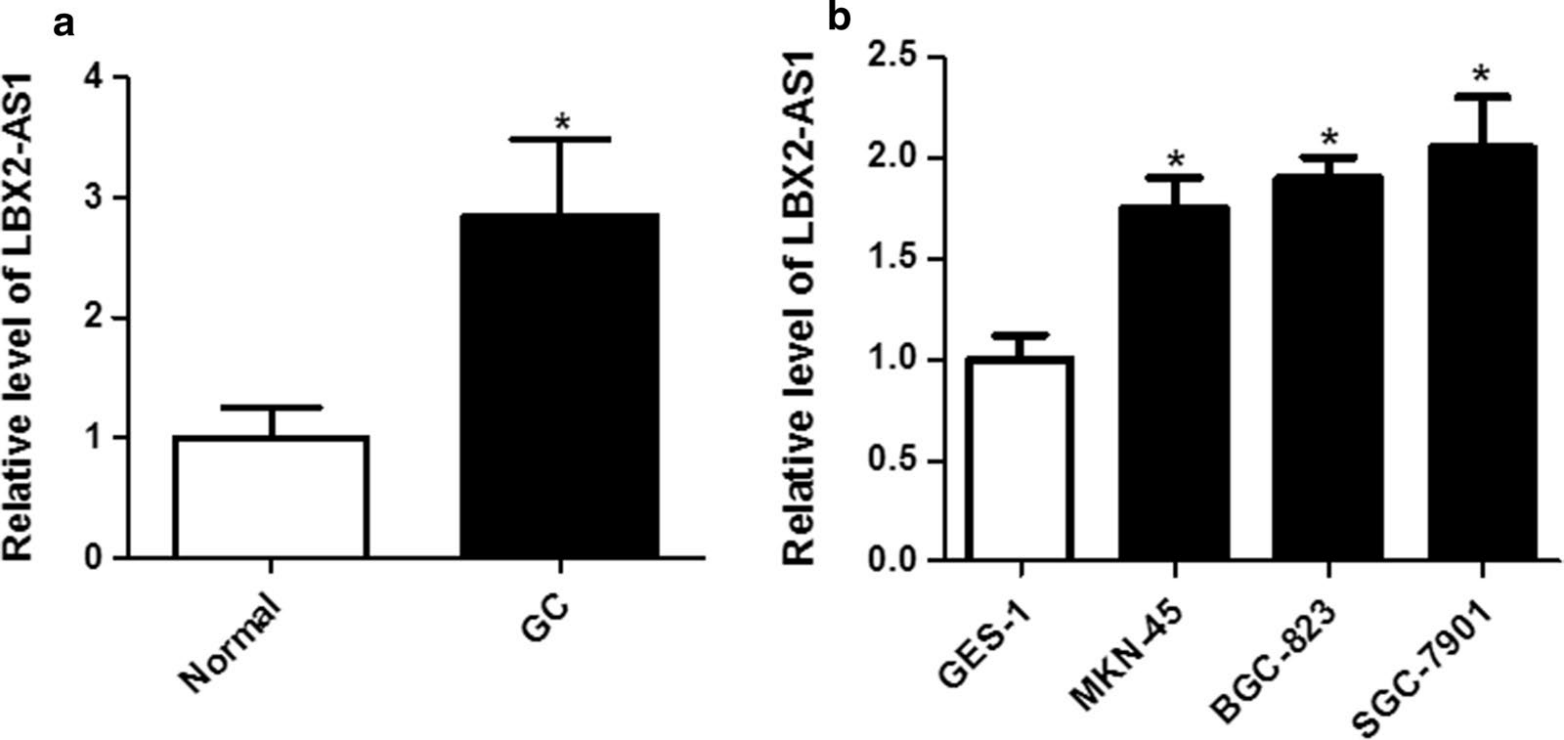
LBX2-AS1 was highly expressed in GC tissue and cells. **a** The expression of LBX2-AS1 was detected by qRT-PCR in GC tissues and adjacent normal tissues. **b** The expression of LBX2-AS1 in cells was detected using qRT-PCR. *P < 0.05 compared with the normal or GES-1 group

In the first, we detected the expression of LBX2-AS1 in GC tissues and cells. As shown in Fig. 1a, LBX2-AS1 level was significantly increased in 78 cases of GC tissues compared with the adjacent normal tissues (*P* < 0.05). Besides, LBX2-AS1 expression in three GC cells (MKN-45, BGC-823 and SGC-7901) was also significantly up-regulated compared with the human gastric mucosal epithelial cell line (GES-1) (Fig. 1b, P < 0.05, respectively).

### LBX2-AS1 knockdown suppressed the proliferation, migration and invasion of GC cells

**Fig. 2:**
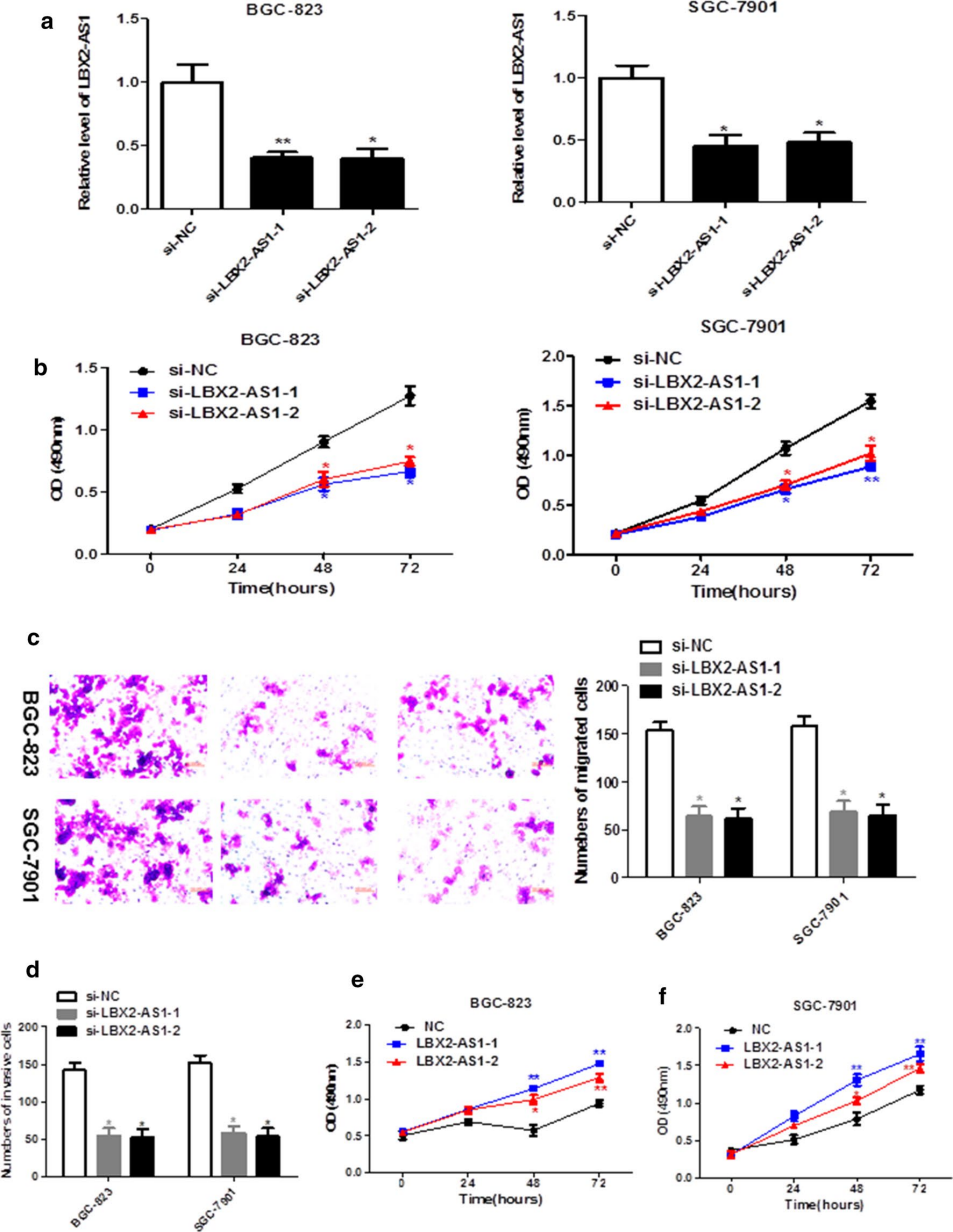
LBX2-AS1 knockdown suppressed the proliferation, migration and invasion of GC cells. **a** QRT-PCR was used to test the LBX2-AS1 level in GC cells after transfected with specific siRNAs. **b** CCK-8 assay was conducted to assess the proliferation ability of GC cells. **c**, **d** Transwell assays were conducted to verify suppressive effects of silenced LBX2-AS1 on the migration and invasion of GC cells. **e**, **f** Overexpression of LBX2-AS1 on the proliferation of BGC-823 and SGC-7901 cells. *P < 0.05, **P < 0.01 compared with the si-NC group

Subsequently, siRNA was used to further explore the role of LBX2-AS1 in the progress of GC. As shown in Fig. 2a, qRT-PCR assay validated that the LBX2-AS1 expression in BGC-823 and SGC-7901 cells was significantly down-regulated when transfected of si-LBX2-AS1, and the si-LBX2-AS1 was chose for the further loss-of-function experiments. Next, CCK-8 assay showed that si-LBX2-AS1 significantly inhibited cell proliferative ability in BGC-823 and SGC-7901 cells (Fig. 2b). Moreover, we found that migrated and invasive of cells were significantly inhibited after knockdown of LBX2-AS1. Statistical results showed that knockdown of LBX2-AS1 strikingly decreased migration and invasion rate of BGC-823 and SGC-7901 cells compared with si-NC-transfected cells (Fig. 2c and d).

### LBX2-AS1 overexpression increased the proliferation of GC cells

As shown in Fig. 2e and f, CCK-8 assay indicated that OV-LBX2-AS1 significantly increased cell proliferation in BGC-823 and SGC-7901 cells.

### LBX2-AS1 directly interacted with miR-4766-5p

**Fig. 3:**
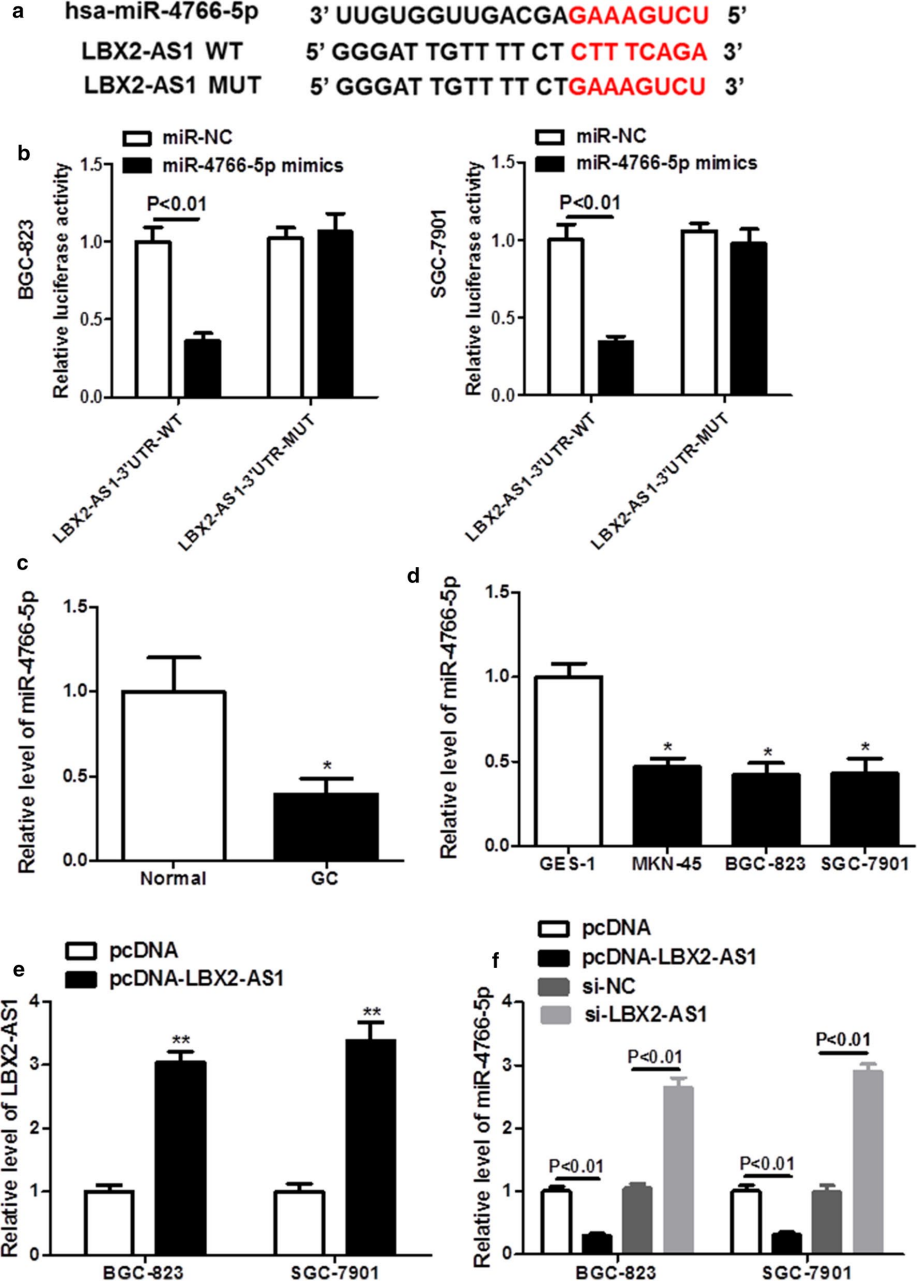
LBX2-AS1 directly interacted with miR-4766-5p. **a** The binding sites between LBX2-AS1-WT/MUT and miR-4766-5p were obtained. **b** Dual luciferase reporter assay was carried out to test the luciferase activity of LBX2-AS1-WT/MUT after cells were transfected with miR-4766-5p mimics or miR-NC. **c** The expression level of miR-4766-5p was examined in GC tissues and non-tumorous tissues with qRT-PCR analysis. **d** qRT-PCR analysis was performed to examine the expression level of miR-4766-5p in cells. **e** The expression of miR-4766-5p was detected in GC cells treated by LBX2-AS1 or si-LBX2-AS1. *P < 0.05 compared with the Normal, or pcDNA group

In order to explore the miRNA associated with LBX2-AS1, we conducted bioinformatics prediction. Analysis indicates that LBX2-AS1 was predicted to contain binding sites of miR-4766-5p (Fig. 3a). And luciferase reporter assay was used to further confirm the prediction. As shown in Fig. 3b, the luciferase reporter assay showed that the luciferase activity markedly reduced when co-transfected with the miR-4766-5p mimic and LBX2-AS1-WT, whereas co-transfection with miR-4766-5p mimic and LBX2-AS1-MUT had no significant effect. Moreover, the expression of miR-4766-5p was found to be significantly reduced in GC tissues and cells compared with the normal tissues and cells (Fig. 3c and d). In addition, LBX2-AS1 expression was notably increased in GC cells after transfected with LBX2-AS1 overexpression plasmid (Fig. 3e), and overexpression or knockdown of LBX2-AS1 significantly reduced or increased the expression of miR-4766-5p in GC cells (Fig. 3f). Taken together, these results showed that LBX2-AS1 directly bind with miR-4766-5p.

### The depletion of miR-4766-5p abrogated LBX2-AS1 deficiency-mediated anti-proliferation, anti-migration and anti-invasion effects in GC cells

**Fig. 4:**
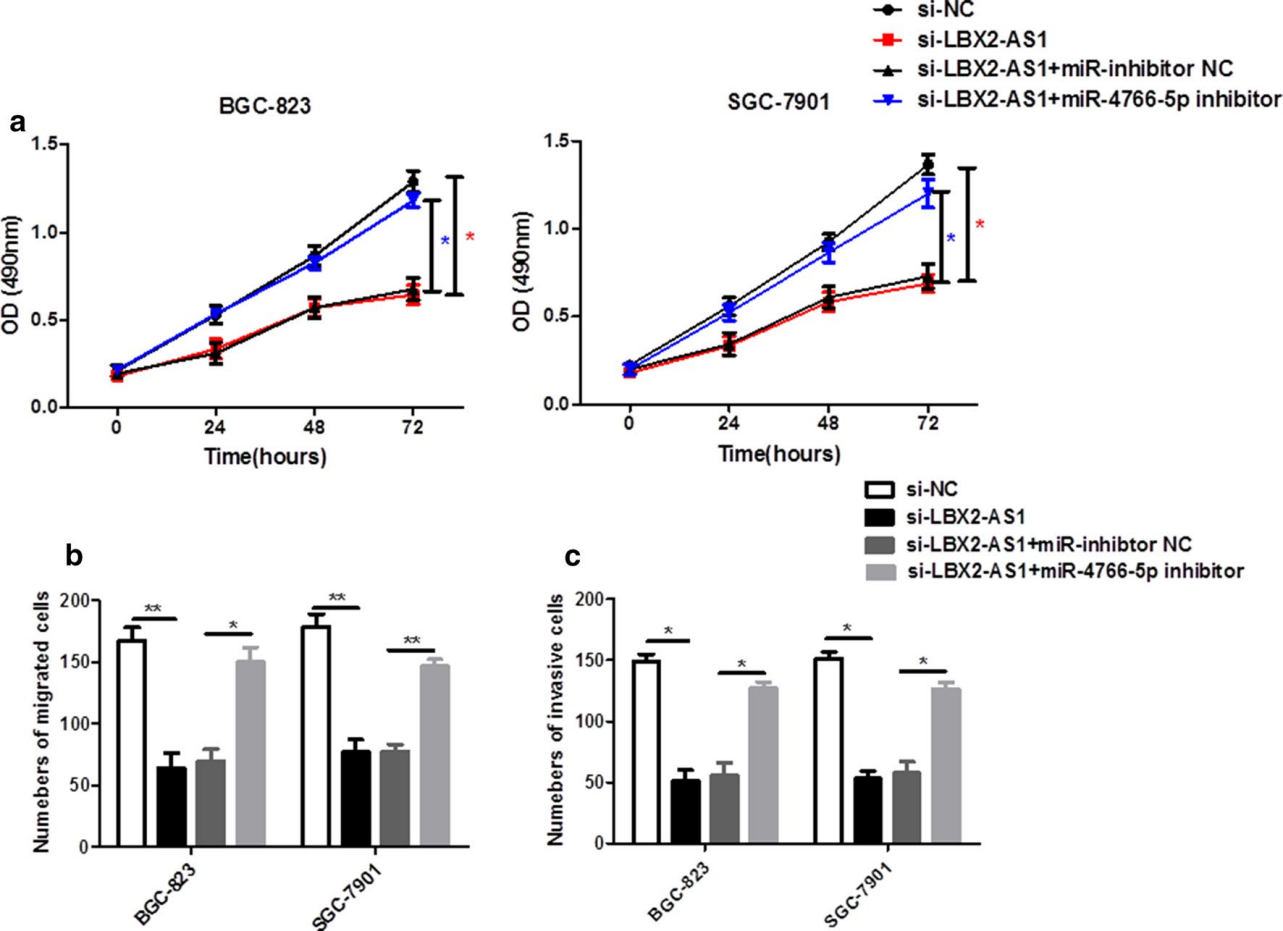
The depletion of miR-4766-5p abrogated LBX2-AS1 deficiency-mediated anti-proliferation, anti-migration and anti-invasion effects in GC cells.**a** CCK-8 assay was used to evaluate the proliferation ability of BGC-823 and SGC-7901 cells after transfection of si-NC, si-LBX2-AS1, si-LBX2-AS1 + miR-NC and si-LBX2-AS1 + miR-4766-5p inhibitor. **b**, **c** Transwell assays were applied to detect the migration and invasion of BGC-823 and SGC-7901 cells after the same transfections. *P < 0.05, **P < 0.01

To verify the effect of LBX2-AS1 and miR-4766-5p expression on cell proliferation, migration and invasion, si-NC, si-LBX2-AS1, si-LBX2-AS1 + miR-inhibitor-NC or si-LBX2-AS1 + imiR-4766-5p inhibitor were transfected into the cells, respectively. As shown in Fig. 4a, blocking the miR-4766-5p led to a remarkable increase of cell proliferation ability in LBX2-AS1 silenced BGC-823 and SGC-7901 cells Moreover, the number of migrated and invasive cells was significantly increased in si-LBX2-AS1-transfected BGC-823 and SGC-7901 cells following the inhibition of miR-4766-5p level (Fig. 4b and c). In conclusion, these data suggested that downregulation of miR-4766-5p significantly inhibited the anti-proliferation, anti-migration and anti-invasion of LBX2-AS1-mediated GC cells.

### CXCL5 was a target of miR-4766-5p

**Fig. 5:**
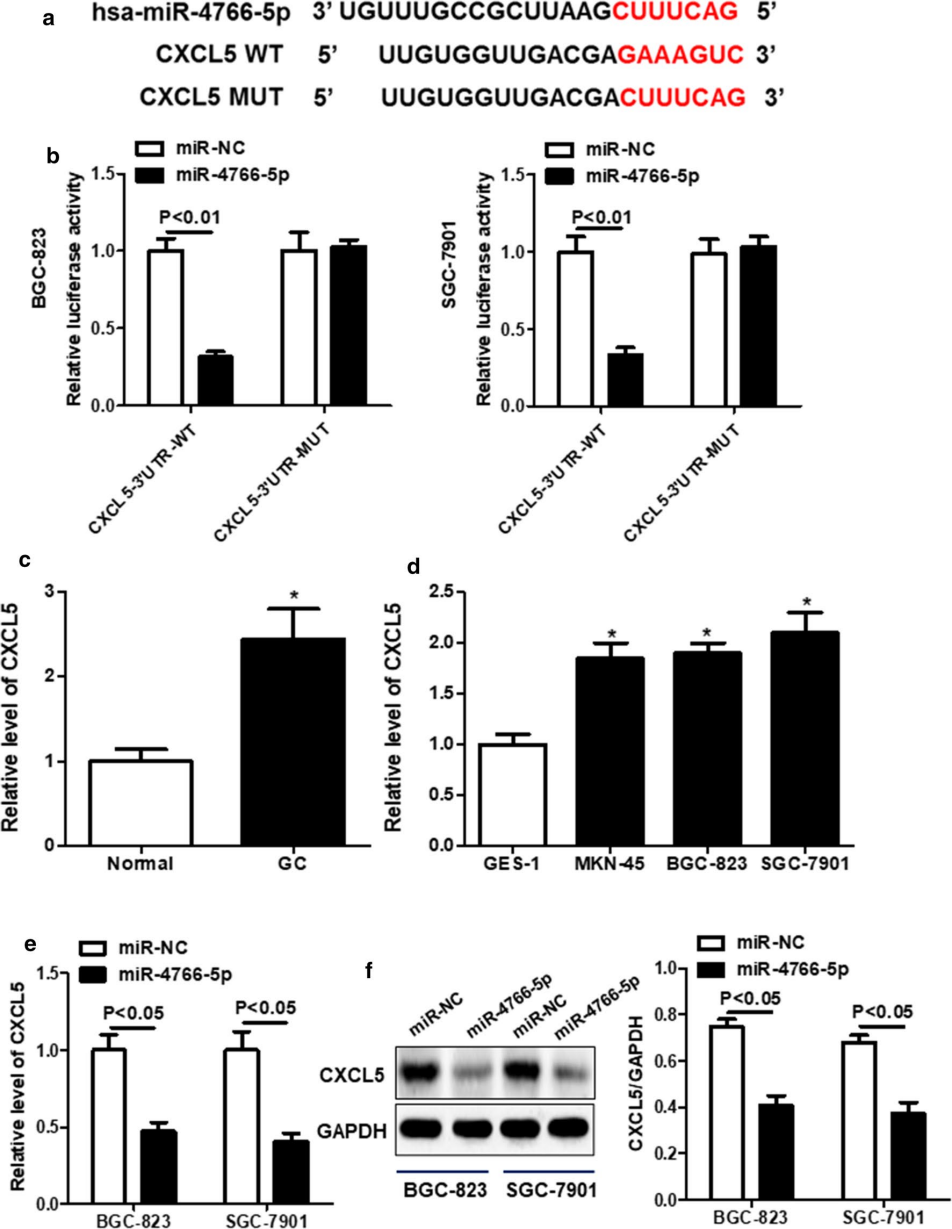
CXCL5 was a target of miR-4766-5p. **a** The binding sites between miR-4766-5p and CXCL5 were shown. **b** Dual luciferase activity of CXCL5-WT/MUT was tested in GC cell lines by performing dual luciferase reporter assays after transfected with miR-4766-5p mimics or miR-NC. **c** The expression of CXCL5 was examined with qRT-PCR in normal tissues and tumor tissues. **d** The mRNA level of CXCL5 was examined in GC cells through performing qRT-PCR. **e** The protein level of CXCL5 was examined in GC cells through performing western blot analysis. *P < 0.05 compared with the Normal, GES or miR-NC group

Bioinformatics prediction revealed that there existed several complementary sequence between miR-4766-5p and CXCL5 3’UTR (Fig. 5a). Dual luciferase reporter assay further showed that the luciferase activity of CXCL5-WT reporter was notably reduced in miR-4766-5p overexpressed cells, while there had no effect on luciferase activity of CXCL5-MUT reporter (Fig. 5b), implying the specificity of miR-4766-5p and CXCL5 3′UTR binding. qRT-PCR assay further revealed that CXCL5 expression in GC tissues was dynamically up-regulated compared with adjacent normal tissues (Fig. 5c). The expression of CXCL5 in three GC cells was significantly increased compared with that in GES-1 cells (Fig. 5d). Moreover, we detected CXCL5 mRNA and protein expression by overexpression of miR-4766-5p, indicating that CXCL5 level was remarkably reduced in miR-4766-5p-overexpressed BGC-823 and SGC-7901 cells (Fig. 5e and f). These data revealed that CXCL5 was a target of miR-4766-5p.

### CXCL5 reversed the inhibition effect of miR-4766-5p and promoted the proliferation, migration and invasion of GC cells

**Fig. 6:**
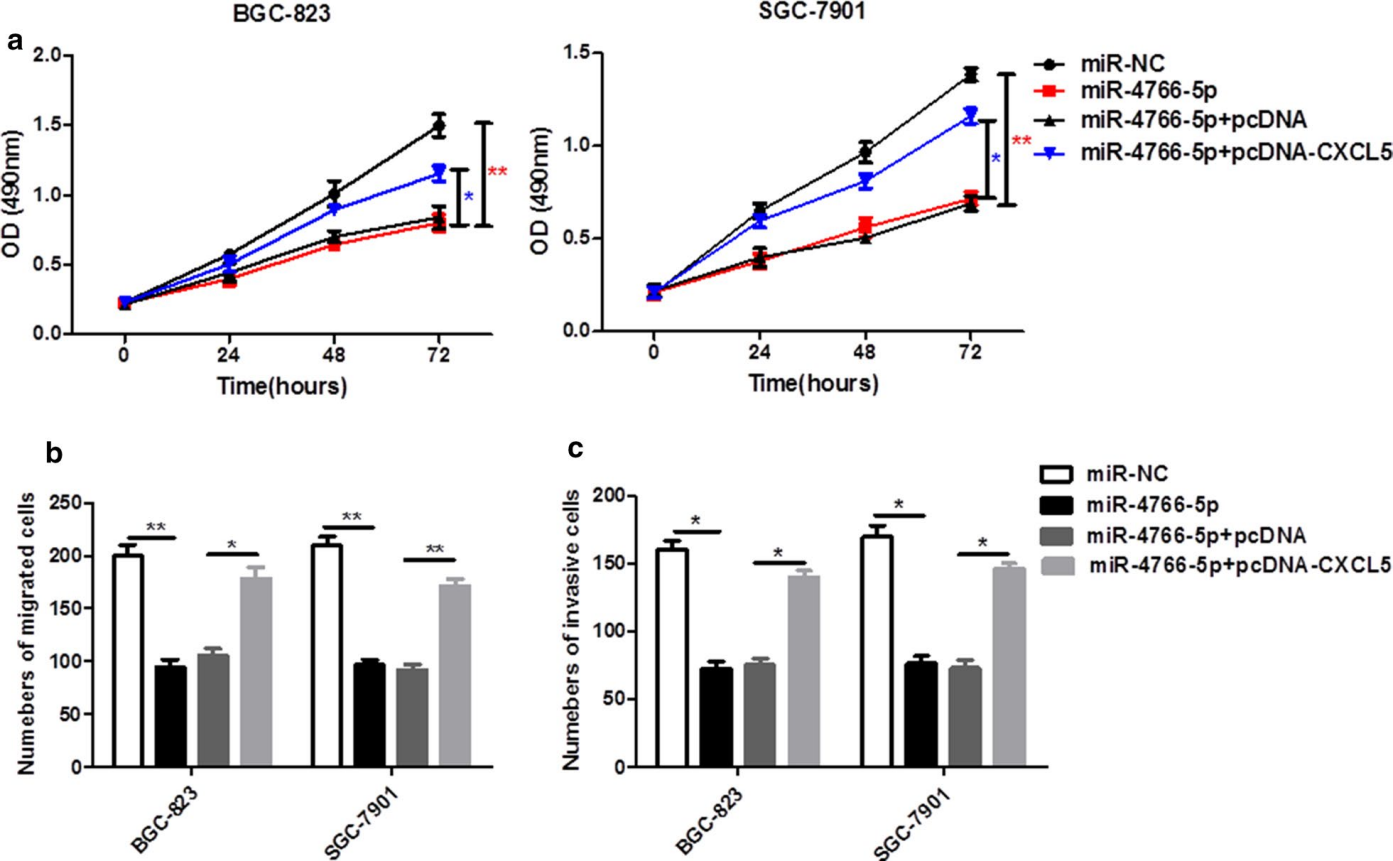
CXCL5 reversed the inhibition effect of miR-4766-5p and promoted the proliferation, migration and invasion of GC cells.  **a** CCK-8 assay evaluated the proliferation ability of BGC-823 and SGC-7901 cells after transfection of miR-NC, miR-4766-5p, miR-4766-5p + pcDNA, miR-4766-5p + CXCL5. **b**, **c** Transwell assays were applied to detect the migration and invasion of BGC-823 and SGC-7901 cells after the same transfections. *P < 0.05, **P < 0.01

To further verify whether miR-4766-5p affect cell proliferation, migration and invasion of GC cells by regulating CXCL5, BGC-823 and SGC-7901 cells were transfected with miR-NC, miR-4766-5p mimics, miR-4766-5p mimics + pcDNA or miR-4766-5p mimics + CXCL5, respectively. CCK-8 showed that the addition of miR-4766-5p mimics significantly inhibited the proliferation ability of cells, while the proliferation ability was restored after the addition of CXCL5 (Fig. 6a). The overexpression of miR-4766-5p significantly inhibited the migration and invasion rate of GC cells, while the addition of CXCL5 restored the migration and invasion of GC cells (Fig. 6b and c). The results indicated that CXCL5 significantly promoted the proliferation, migration and invasion of GC cells.

### LBX2-AS1 promoted CXCL5 expression by relieving miR-4766-5p-mediated inhibitory effect on CXCL5 in GC cells

**Fig. 7:**
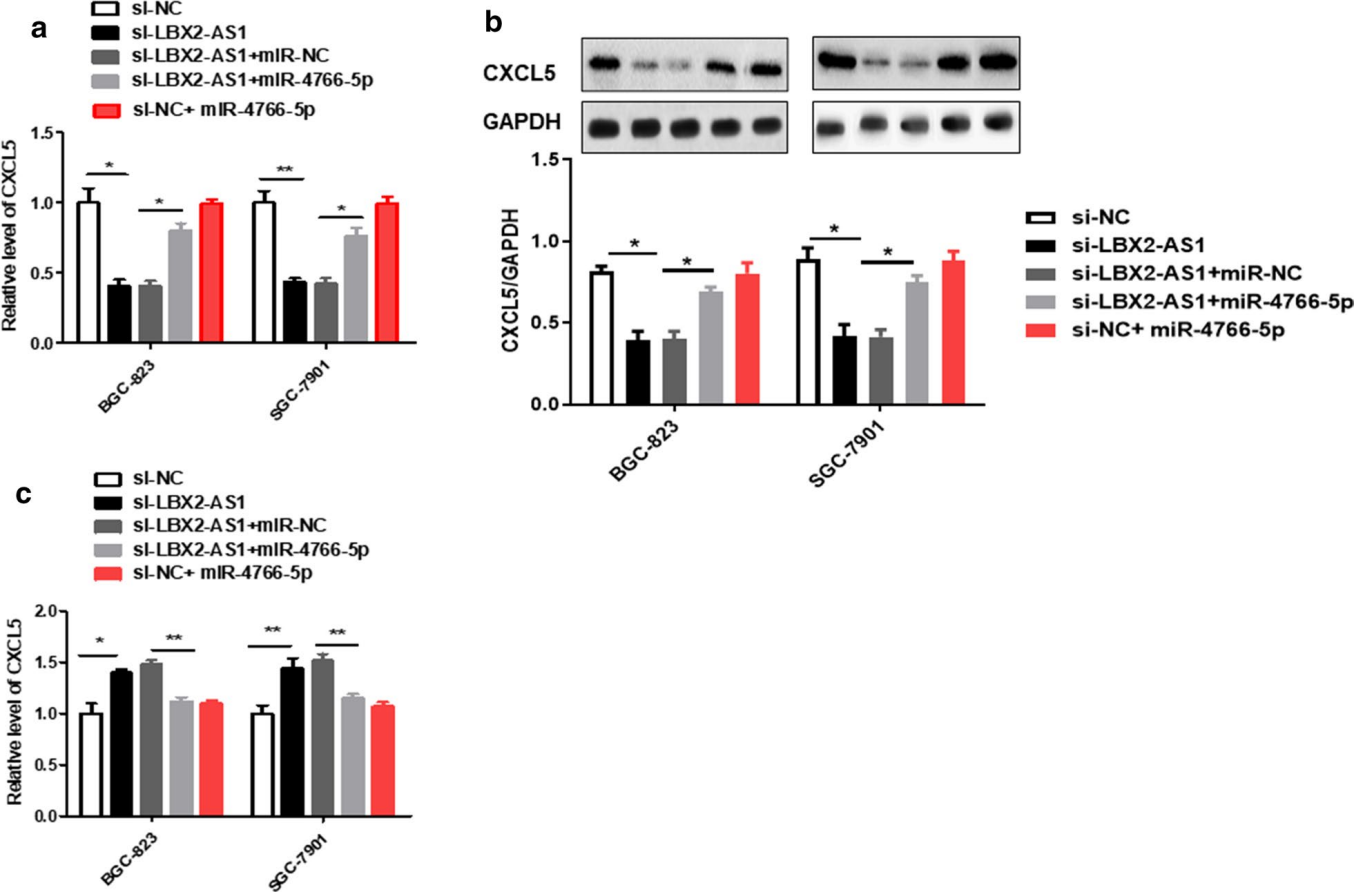
LBX2-AS1 promoted CXCL5 expression by relieving miR-4766-5p-mediated inhibitory effect on CXCL5 in GC cells. **a** The mRNA level of CXCL5 was examined in GC cells after transfection of si-NC, si-LBX2-AS1, si-LBX2-AS1 + miR-NC and si-LBX2-AS1 + miR-4766-5p inhibitor through performing qRT-PCR. **b** The level of CXCL5 protein was tested with western blot assay after the same transfection. *P < 0.05, **P < 0.01

Based on the above research, we further detected CXCL5 mRNA and protein levels with treated knockdown of LBX2-AS1 or inhibition of miR-4766-5p, in order to verify the relationship between the three. CXCL5 mRNA level was dynamically down-regulated in LBX2-AS1-silenced GC cells, however, miR-4766-5p inhibitor abolished LBX2-AS1 knockdown-mediated CXCL5 downregulation in BGC-823 and SGC-7901 cells (Fig. 7a). The results of protein levels were consistent with mRNA levels (Fig. 7b). While, LBX2-AS1 overexpression increased CXCL5 mRNA up-regulation in BGC-823 and SGC-7901 cells (Fig. 7c).

### RNA pull down in BGC-823 and SGC-7901 cells

**Fig. 8:**
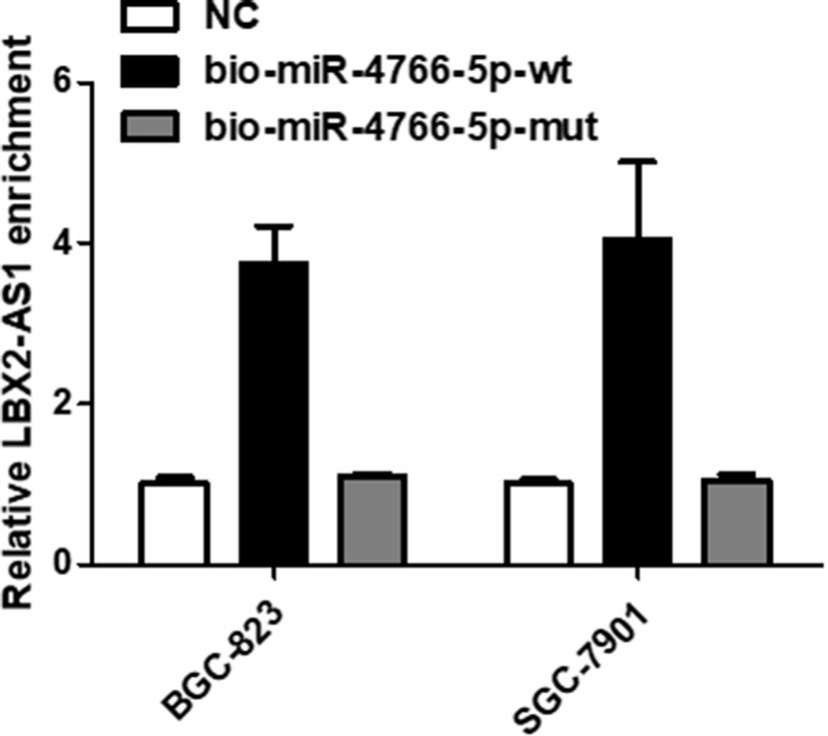
RNA Pull down in BGC-823 and SGC-7901 cells. The specificity of LBX2-AS1 and miR-4766-5p was examined by RNA pull down

As shown in Fig. 8, in order to study the specificity of LBX2-AS1 and miR-4766-5p, the RNA pull down experiment was used to verify the specificity of LBX2-AS1 and miR-4766-5p on BGC-823 and SGC-7901 cells. BGC-823 and SGC-7901 cells, miR-4766-5p specifically binds to LBX2-AS1.

## Discussion

GC is a malignant tumor that originates from the gastric mucosal epithelium, and has the highest incidence among various malignant tumors in China [17]. Recently, more and more papers indicated that GC patients still suffer a diagnosis in late stage and a high mortality [18]. With the rise of studies on the functional properties of lncRNAs, it has been verified that lncRNAs efficiently affect biological processes of cancers, and exerts pivotal properties [19, 20], and process important roles in human tumors including GC. *In vivo* experiments showed that the expression of LBX2-AS1 contributed to the occurrence of tumors. This suggests that LBX2-AS1 may be involved in tumor development as a tumor promoter.

In terms of mechanism, lncRNA can be used as competing endogenous RNA (ceRNA) sponge cells and play different functions together with target miRNA. LBX2-AS1 was an overexpressed lncRNA in nearly 70% of cancers, implying its huge potential in tumor regulation. In our study, bioinformatics predicted that LBX2-AS1 had a miR-4766-5p binding site, and further experimental verification indicated that miR-4766-5p functioned as a tumor suppressor in GC tissues and cells, this is consistent with previous studies on the role of miR-4766-5p in breast cancer [9]. Inhibition of miR-4766-5p can reverse the effect of LBX2-AS1 knockout on cell anti-proliferation, anti-migration and anti-invasion. This is consistent with the results of Wei et al. study that miR-4766-5p inhibited growth, migration and invasion in GC [21]. Our findings demonstrated that miR-4766-5p was a downstream target of LBX2-AS1 in GC.

Many studies have shown that CXCL5 promotes cancer progression. Miyazaki et al. study showed that CXCL5 production results in increased proliferation and invasion in squamous cell carcinomas [22]. Ma et al. showed that CXCL5 promotes hepatocellular carcinoma cell proliferation, invasion, and intratumoral neutrophil infiltration [23]. Moreover, colorectal cancer cells has been reported to secret CXCL5, which could promote cell proliferation and migration as well as invasion [24]. In our study, CXCL5 was proved to be a target of miR-4766-5p and positively related to LBX2-AS1. Meanwhile, LBX2-AS1 knockdown was able to inhibit CXCL5 mRNA and protein expression, which can be reversed by miR-4766-5p inhibitor. CXCL5 improved cell proliferation, migration and invasion of GC cells. Overexpression of CXCL5 overturned these effects of LBX2-AS1 knockdown on proliferation, migration and invasion in GC cells.

## Conclusions

Taken together, our study showed that LBX2-AS1 promoted proliferation, migration and invasion via downregulating miR-4766-5p and upregulating CXCL5 in GC cells. This provides some potential therapeutic targets for GC research and provides a basis for us to explore the role of lncRNA-miRNA functional network in cancer. All these findings might actually provide new therapeutic opportunities of GC for this newly identified regulatory signaling pathway.

## Abbreviations

LBX2-AS1: LBX2 antisense RNA 1
LncRNA: Long noncoding RNA
GC: Gastric cancer
CXCL5: C-X-C motif chemokine
qRT-PCR: Quantitative real-time polymerase chain reaction
CCK-8: Cell counting kit-8
siRNA: Small interfering RNA

## Availability of data and materials

The data used to support the findings of this study are included in the article.
